# Mange in Rabbits: An Ectoparasitic Disease with a Zoonotic Potential

**DOI:** 10.1155/2022/5506272

**Published:** 2022-07-16

**Authors:** Wafaa A. Abd El-Ghany

**Affiliations:** Poultry Diseases Department, Faculty of Veterinary Medicine, Cairo University, Giza 12211, Egypt

## Abstract

Mange in rabbits is a very important parasitic disease causing high losses. The disease is caused mainly by *Sarcoptes scabiei*, *Psoroptes cuniculi*, *Cheyletiella parasitovorax*, and *Notoedres cati*. Body mange and ear mange are the most common forms of this disease in rabbits. Animals can get mite infestation through direct contact with infected animals or contaminated fomites. This infestation is characterized by zoonotic nature and public health burden. The skin affection is characterized by pruritus, alopecia, severe cachexia, and sometimes death. Infestation is diagnosed mainly by skin scraping and microscopic examination. Control measures mainly depend on the use of different types of systemic and topical acaricides and the use of natural products and supportive elements. Vaccine is not commercially available and is still under investigation. Accordingly, this review article was designed to shed the light on the mange disease in rabbits in terms of mite's infestation and susceptibility, clinical manifestations, zoonosis, diagnosis, and control strategies.

## 1. Introduction

Rabbits have been regarded as key livestock, which are increasingly being raised in many countries worldwide [1]. These animals have been intentionally released as a source of meat and fur production [2]. However, diseases are the major challenges facing the sustainability of rabbit farming.

**Figure 1:**
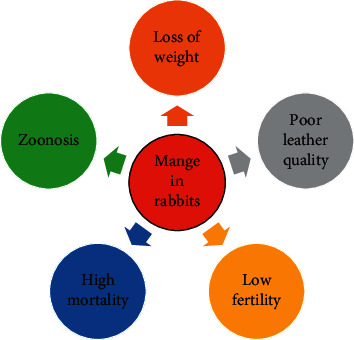
Economic losses in rabbit's production system due to manage infestation.

Mange in rabbits is an emerging and highly contagious disease caused by different burrowing and non-burrowing mite species protozoon [3]. Mange affects not only rabbits for meat production but also free-ranging wild populations [4], with deleterious consequences for rabbit population viability [5]. Mites induce several skin conditions in rabbits such as psoroptic, sarcoptic, and notoedric mange [6]. Ectoparasites including *Sarcoptes scabiei* var. *cuniculi*, *Psoroptes cuniculi*, and *Cheyletiella parasitovorax* are the causes of mange in rabbits. Moreover, *Notoedres cati cuniculi* burrowing mite has been detected in rabbits [2, 7, 8]. However, *Psoroptes ovis* has been identified in rabbits with ear lesions [9–11]. In 2017, scabies has been added to the World Health Organization Neglected Tropical Diseases portfolio [12]. Mange infestation is one of the major constraints affecting commercial rabbit flocks [13, 14]. This disease causes severe economic losses in the production system of rabbits due to loss of productivity, poor leather quality, decreasing conception rates, loss of weight, and high mortalities [15, 16] (Figure 1). Mange is characterized by pruritus, alopecia, and prolonged illness with severe cachexia [17]. In addition, mite infestation is associated with vestibular dysfunction and meningitis [18]. Pruritic and skin lesions are formed as a result of the parasites feeding on the stratum granulosum of epidermis and animal's serum [19]. Blood loss and frequent complications with secondary bacterial infections may be expected after mite infestation [20]. The anthropozoonosis importance of mites cannot be ruled out [21]. Globally, more than 100 million persons suffer from mite infestation [22].

Accordingly, this review article was designed to give a focus on mange disease in rabbits and direct a spotlight upon mite's infestation and susceptibility, clinical manifestations, zoonosis, diagnosis, and control strategies.

## 2. Infestation and Susceptibility

The adults, nymphs, and larvae of the parasite can infest the host. Also, mites can sense the host's temperature and smell [23]. The larvae are highly pathogenic where they infest the healthy host via direct contact with the skin in less than 20 minutes. The whole life cycle takes more than one month [24]. Females oviposit in the tunnels of the skin's stratum corneum, inducing hypersensitivity, inflammation, and skin rashes. After 3–10 days, larvae are hatched and moved on the skin searching for hair follicles, moulted, and then matured into adult mites. The adult mites live from 3 to 4 weeks in the host's skin.

The main route of infection and transmission of mites is the direct contact between infested and healthy rabbits or indirect contact with contaminated fomites or environment [25]. Overcrowding and poor hygiene are significant factors for mite infestation [19]. The disease is common in subtropical countries, especially during the rainy and winter seasons due to the low temperature and high humidity [26, 27]. *Notoedres cati* is likely to be transmitted from infested cats to rabbits and vice versa [6, 28]. Sometimes, the rabbits reared out of the house were suggested to get mite infestation from domestic cats. Moreover, wild animals may transmit sarcoptic mange, either naturally or experimentally, from dogs to rabbits or rabbits to dogs [29]. Kids could be infested during the suckling from adult-infested rabbits [30].

All ages and both sexes of rabbits are susceptible to mite infestations [2]. However, severe infestation with high mortalities has been observed in young and debilitated animals [31]. Moreover, Elshahawy et al. [2] demonstrated a higher incidence of mite infestation in young rabbits compared with adult ones. All breeds of rabbits can get mite infestation. However, some breeds are more susceptible than others, which may be explained by the genetic differences among breeds. Rabbits in poor conditions appear to be more susceptible than others [32].

## 3. Clinical Pictures

The clinical pictures of different mite species infesting rabbits are listed in Table 1. Infestation of rabbit's ear with *Psoroptes cuniculi* may cause typical psoroptic, otoacariasis, or ear canker disease. The infection is not limited to the ear canal and pinnae, but it may extend extra-auricular and spread over the rabbit's body [41, 42]. The parasite lives in the external auricular meatus where it is fed on skin secretion, serous exudate, and blood [33].

However, deep burrowing and chewing *Sarcoptes scabiei* var. *cuniculi* mites can invade rabbits' skin causing tunnels [13]. These skin lesions are attributed to (i) direct mechanical stimulation of mites and mucus from mange, (ii) allergic reaction that is created by immediate or delayed sensitivity reaction, and (iii) mechanical damage due to excessive itching and rubbing to objects. Moreover, affected animals manifest pyodermatitis due to allergic irritation and damage to the skin or extracellular products of mite such as interleukin-1 [37]. The presence of a large number of parasites below the crusts is related to weak hypersensitivity reaction of rabbits [43]. Infested rabbits reveal anorexia as a result of painful sensation of chewing.

Other species of mites such as *Cheyletiella parasitovorax* [39], *Notoedres cati cuniculi* [27, 44], and *Psoroptes ovis* [10, 11] have been also identified in rabbits with skin lesions.

## 4. Zoonosis and Public Health Burden

Scabies is widely distributed in populations all over the world [45], especially in the developing countries [46, 47]. It is listed as a neglected tropical disease [48]. Scabies is a significant human public health threat with a financial burden [49]. This disease may be associated with secondary complications in humans such as pyoderma [50], rheumatic heart disease, and acute post-streptococcal glomerulonephritis [51]. Children are more susceptible to scabies than adults [22]. The zoonotic transmission possibility of mite infestation has been previously reported [52]. Humans may be infested after direct contact with infected animals [53]. For instance, a reported case of human scabies showed a history of direct contact with his pet dog [54].

## 5. Diagnosis

The severity of mite infestation clinical picture is classified as “absent” if skin abnormality is not detected; “mild” if the severity of signs is low along small-area lesions in the body; “moderate” if the intensity of manifestation is excessive over a small skin area or with low intensity over a large skin area; and “severe” if the manifestation is of great intensity over a large area of the body. The examined areas of the body are ears, head, neck, thorax, abdomen, and extremities. In ear mange, the lesion score was as follows: 0 for apparently normal ears; 1 for lesions inside the ear; 2 for lesions on the bottom third of the ear; 3 for lesions extending to the two thirds of the ear; and 4 for lesions with a greater extension than two thirds of the ear [9].

The main method of mite laboratory diagnosis is the skin scrapings of lesion edges in suspected animals [55]. A lesion with a diameter area of 2.5 cm^2^ is moisturized with mineral oil and scrapped at the periphery with a sharp, clean, and sterilized scalpel till oozing of the blood. The scrapping should be collected, put in tubes containing potassium hydroxide (10%), heated in a water path at 60–80°C for 15 min, and then centrifuged at 1500–2000 rpm for 5 min. The supernatant is removed, some drops of sediment are placed on a glass slide, and permanent mounts of the parasite should be prepared [56]. Mite species could be determined through the morphological characteristics under a microscope [57, 58]. Sometimes, epidermal debris and hair could be collected in a Petri dish and directly examined using the stereomicroscope. Adult *Sarcoptic scabiei* is round with short legs, having a long unjointed stalk with a sucker on the front pair of legs. The dorsal body surface of sarcoptic mite has a thick and chitinous wall with large spines. The anus is terminal, and the dorsum possesses scales, cones, and bladelike setae [59].

Sarcoptes-infested rabbits showed altered antioxidant systems to states of oxidative stress [60]. Blood antioxidant parameters such as glutathione peroxidase, superoxide dismutase, total antioxidant capacity, and thiobarbituric acid-reactive substances were increased after mite infestation as an index of lipid peroxidation [61]. The level of malonyldialdehyde in mite-infested rabbits was markedly increased as it was associated with skin cell deterioration and the development of skin lesions [62]. In addition, the levels of total protein, albumin, globulin, cholesterol, triglyceride, high- and low-density lipoprotein, aspartate aminotransferase, and alanine aminotransferase activities were also other parameters that could be taken into consideration during mite infestation of rabbits [61, 63].

Serological tests such as enzyme-linked immunosorbent assay are used to detect the antibodies against *Sarcoptes scabiei* [64, 65].

Since conventional diagnostic techniques for mite diagnosis have less than 50% accuracy [66], confirmatory diagnosis through molecular techniques became essential. Confirmation of mite species could be obtained via the amplification of a specific fragment [67]. Polymerase chain reaction (PCR) is regarded as a highly sensitive, reliable, and specific method for mite identification [55].

## 6. Control Strategies

**Figure 2:**
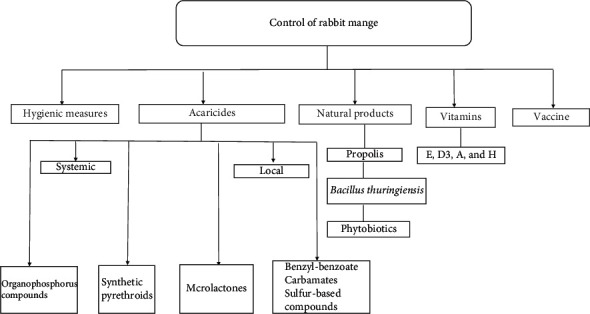
Different strategies to control mange in rabbits.

Many strategies have been applied to control mange in rabbits (Figure 2) including the application of hygienic measures and the use of acaricides, natural products, and vaccines [68]. Thorough disinfection of rabbit cages and surroundings is very critical for effective control of mite infestation [7].

Effective treatment of mange in rabbits could be achieved through using some specific parasiticides or acaricides. Organophosphorus compounds (diazinon), synthetic pyrethroids (deltamethrin and permethrin), and macrolactones (ivermectin derivatives) are the most commonly used acaricides. Avermectin derivative group includes ivermectin [14, 69–71], eprinomectin [9, 18, 72], doramectin [73, 74], selamectin [75–77], moxidectin [58, 78], and abamectin. Moreover, benzyl benzoate, carbamates, sulfur-based compounds [79, 80], and paraffin oil [71] may be used also for mange treatment. For example, ivermectin, carbaryl, and liquid paraffin proved efficacy against psoroptic and sarcoptic infestations of rabbits [81]. In addition, Divisha et al. [82] concluded that the isolation of diseased rabbits, treatment with ivermectin, topical application of benzyl benzoate, and carrying out management practices could effectively control mange in rabbits.

### 6.1. Chemical Acaricides

#### 6.1.1. Ivermectin

Ivermectin opens the glutamate-gated and gamma amino-butyric acid-gated chloride channels, which block the transmission of signals from the central interneurons to peripheral neurons of mite. Thus, the resistance of muscle's membrane is decreased with hyper-polarization of cells, and the parasite shows paralysis and finally dies [83]. The drug has proven broad-spectrum efficacy against ectoparasites and endoparasites in most animal species. The efficacy of ivermectin in the treatment of mange in rabbits is well established globally [8, 84]. The highly achieved efficacy of ivermectin in the keratin layer might be due to the high concentration of the drug in the skin [85]. Ivermectin is usually administered to rabbits using subcutaneous route. Kaya et al. [74] found that ivermectin induced more rapid effect than doramectin in the treatment of *Sarcoptes scabiei* in rabbits. Administration of ivermectin at a dose of 0.2–0.4 mg/kg body weight (b/wt) once every 2 weeks for 2-3 times [86], 0.2–0.4 mg/kg·b/wt once every 2 weeks for 2-3 times [87], and 400 *μ*g/kg·b/wt for 3 weeks at weekly intervals [88], and 700 mcg/kg·b/wt [89] was very efficient in controlling *Psoroptes cuniculi* and *Sarcoptes scabiei* in rabbits. Besides, Isingla et al. [90] reported complete cure of rabbits from Notoedres mange with negative skin scrapings after 20 days of ivermectin therapy. Moreover, *Sarcoptes scabiei*-infested rabbits showed recovery after 4 successive ivermectin treatments at a dose of 200 *μ*g/kg·b/wt at weekly intervals with supportive therapy using pheniramine maleate [1]. Nearly similar recent results were obtained by Narang et al. [44] who demonstrated that a single dose of ivermectin (400 *μ*g/kg·b/wt) combined with chlorpheniramine maleate (0.4 mg/kg·b/wt) and topical liquid povidone-iodine twice a day helped in the complete cure of *Notoedres cati* var. *cuniculi*-infested rabbits. The authors suggested prolonged availability of ivermectin with a residual effect against mites in rabbits. Complete cure from mite infestation after 7 days of ivermectin treatment is suggestive of the availability of sufficient drug concentration to kill any larvae hatching from the eggs. Ivermectin may cause the release of free radicals, which results in cytotoxic effect on the parasite [91].

However, ivermectin may show some disadvantages such as the genotoxic and cytotoxic effects [92]. Moreover, the resistance of *Sarcoptes scabiei* to ivermectin has been developed [93] along with environmental pollution [94]. This drug has a negative impact on the reproductive performance of male and female rabbits. El-Nahas and El-Ashmawy [95] demonstrated that ivermectin administration may cause a decrease in the weight of sexual organs, which reflects on the animal's production. It induced necrosis of spermatogenic cells and absence of sperms in males and severe haemorrhages of the uterus and degeneration of atretic follicles and ova in the ovaries of females [92]. Moreover, the liver function tests such as aspartate aminotransferase and alanine aminotransferase were negatively affected after ivermectin injection in rabbits [96].

#### 6.1.2. Selamectin

Selamectin could be used effectively for the treatment of *Psoroptes cuniculi* at a dose of 6 or 18 mg/kg·b/wt once or twice at 28-day intervals [75], or at a dose of 6–18 mg/kg·b/wt as a single application [76]. Moreover, it showed good results against *Sarcoptes scabiei* var. *cuniculi* at doses of 8–14 mg/kg·b/wt once or twice, 30 days apart [77]. Regarding *Cheyletiella* species, selamectin has been efficiently applied once at a dose of 12 mg/kg·b/wt [97] or at doses of 6.2–20.0 mg/kg·b/wt 1–3 times during an interval of 2–4 weeks [98]. Moonarmart et al. [99] revealed that topical application of selamectin at a dose of 15 mg/kg·b/wt at a 2-week interval was effective and safe in the control of *Sarcoptes scabiei* var. *cuniculi*, *Psoroptes cuniculi*, and *Cheyletiella* species for at least 58 days after treatment.

#### 6.1.3. Doramectin

It has been found that doramectin, a genetically modified avermectin, shows a greater half-life time in plasma than that of ivermectin [100]. After parenteral administration, rabbits can absorb and eliminate this drug quicker than other animal species [101]. Parenteral application of different doses of doramectin was carried out for the control of *Psoroptes* species in rabbits with promising results [62]. Ear lesions of rabbits caused by *Psoroptes ovis* were regressed after 14 days of treatment with doramectin injection [102]. A single dose of doramectin at a concentration of 400 *μ*g/kg·b/wt along for 3 days was very efficient against Notoedric mange in rabbits [73]. The efficacy of ivermectin and doramectin was compared in sarcoptes species-infested rabbits, and the results showed equal efficacy but quicker recovery in ivermectin-treated rabbits [74].

#### 6.1.4. Moxidectin

A topical mixture of imidacloprid and moxidectin was a practical and well-tolerated means of eradication of *Psoroptes cuniculi* in rabbits without adverse reactions [58]. Rabbits with psoroptic mites were subcutaneously treated with moxidectin (0.2 mg/kg·b/wt) orally 2 times, with 10 days in between, resulting in the absence of cerumen or mites in the external ear canal where the animals were completely cured during the next 6 months [78].

#### 6.1.5. Carbaryl

Carbaryl, one of the carbamate groups, is a broad-spectrum insecticide [103]. It acts by the inhibition of acetylcholinesterase enzyme, which hydrolyzes acetylcholine into choline and acetic acid, resulting in disrupting the transmission of nerve impulses in the parasite [104]. Carbaryl treatment may be toxic, laborious, and expensive and requires initial wetting of the lesion before use; thus, its application in the control process is limited.

#### 6.1.6. Benzyl Benzoate

Diluted benzyl benzoate is used as a topical treatment of scabies to decrease the severity of skin irritation [105]. A mixture of topical anti-scabietics such as benzyl benzoate, crotamiton, lindane, and permethrin was effective in controlling the disease [106]. However, prolonged administration of benzyl benzoate may cause contact dermatitis.

#### 6.1.7. Sulfur-Based Compounds

Topical sulfurated lime is a mixture of polysulfides of plant origin, which has been used for controlling skin disease of ectoparasite origin via weekly application with dilution of 1 : 16 or 1 : 32 [107].

#### 6.1.8. Synthetic Pyrethroids

Synthetic pyrethroids such as deltamethrin, permethrin, and cyfluthrin were used for long time as efficient acaricides. Permethrin (5%) is recommended by the Centers for Disease Control and Prevention as the first-line topical therapy for scabies [108]. Permethrin has been used effectively to control sarcoptic mite in rabbits and sheep [109]. In the study of Abdelaziz et al. [80], the efficacy of 5% of deltamethrin, 10% of cyfluthrin, and 10% of sulfur ointment was investigated for the treatment of sarcoptic mange in rabbits. The results indicated that all these compounds were effective after the 28th day of treatment; however, deltamethrin was the least efficient drug in sarcoptic mange treatment. Double doses of deltamethrin could eradicate mites in rabbits [110, 111]. An *in vitro* study showed that 5% of permethrin demonstrated 100% effectiveness against mite within 12 hr, while 5% of deltamethrin killed 28% and 32% of mites [42]. Another study by Pap et al. [112] showed the inefficacy of deltamethrin (5%) in controlling *Psoroptes cuniculi*. Cyfluthrin proved to have an efficient pesticidal effect in the elimination of *Sarcoptes scabiei* from the environment [113]. Increasing the resistance to some chemicals such as deltamethrin and flumethrin has been developed with variable levels of cure against mange in rabbits.

#### 6.1.9. Paraffin and Other Topical Insecticides

Some oils such as liquid paraffin may be efficient in mange treatment [114]. Oils make direct contact with the parasite and may also block the opening of stratum corneum through which the buried mites breathe; thus, the mites are suffocated [113, 115].

Fipronil and milbemycin oxime are topical compounds, which have been applied via pouring them on lesions for 2–4 successive weekly doses [116]. In addition, parenteral double administration of ivermectin 200 *μ*g/kg·b/wt, fipronil spray in intervals of 15 days along with oral chlorpheniramine maleate (1 mg/kg·b/wt once in a day), and topical application of 5% of iodine for 5 days were the effective incomplete recovery protocol of rabbits from *Sarcoptes scabiei* after one month of treatment [6].

This treatment is easily applicable, is relatively cheap, has no adverse effects, reduces the possibility of using systemic acaricides, and shows low incidence of resistance and accumulation of edible tissue's residues.

### 6.2. Natural Products

The development of drug resistance, presence of tissue residues, and adverse effects on the health and productivity of rabbits prompted and necessitated the search efforts to discover non-conventional and innovative effective alternative control therapies against mange.

#### 6.2.1. Propolis

Propolis (bee glue) has various biological and pharmacological activities due to the presence of flavonoids, aromatic acids, and esters [117]. It could be used as an efficient and safe natural therapy to control mange and avoid the side effects of chemical drugs [118]. The acaricidal effect of propolis on the body or ear's mite may be attributed to the remarkable reduction in the metabolic rates of parasites. Propolis treatment of rabbits showed a potential restoration of antioxidants' properties and amelioration of lipid peroxidation that accelerates clinical and parasitological cures [118]. The concentration and contact time of propolis on the skin lesions are important factors for enhancing treatment. Topical application of 10% of propolis ointment for 3 successive days resulted in the complete absence of skin lesions [119]. Moreover, the treatment of mite-infested rabbits with either ivermectin (400 *μ*g/kg·body weight) or 10% of propolis ointment induced the reduction in signs and pathological lesions, absence of adult mites and developmental stages, and improvement of some biochemical parameters [118].

#### 6.2.2. *Bacillus thuringiensis*

There are about 60,000 *Bacillus (B) thuringiensis* strains that may have specific insecticidal efficacy [120]. It has been estimated that over 90% of the bio-insecticides market includes compounds containing *B. thuringiensis* [121]. Dunstand-Guzmán et al. [122] concluded that *B. thuringiensis* protein extracts are regarded to be potential biological control of mange in rabbits. The mode of action of *B. thuringiensis* against mites is not well defined; however, alterations in the intestinal columnar epithelial cells after mite infestation may be attributed to the activation of *B. thuringiensis* protoxins such as Cry1A. Cry1A acts through a specific “Receptor A,” which is aminopeptidase-N that has been purified from brush border membranes of intestinal epithelial cells of Lepidoptera [123].

#### 6.2.3. Phytobiotics

Phytobiotics such as plant extracts [124], plant essential oils [125–128], garlic extract [129, 130], and turmeric extract [61, 131] gained much attention for the treatment of mange in rabbits. Emtenan et al. [128] found that rabbits treated with cinnamon oil and infested with sarcoptes revealed an elevation in the total protein, albumin, and globulin when compared to non-treated animals. Garlic or cinnamon oil in a concentration of 5% was better than ivermectin in the treatment of *Sarcoptes scabiei*-infested rabbits without any side effects on the liver and kidney functions or the semen characteristics [63].

### 6.3. Vitamins

Supportive treatment using vitamins with other therapeutic medicaments could improve the clinical recovery of rabbits against *Sarcoptes scabiei* [132]. Some vitamins such as vitamins E, D3, A, and H act as antioxidants and enhance the recovery of *Psoroptes cuniculi*-infested rabbits [60, 62].

### 6.4. Anti-Mite Vaccine

It has been shown that the application of acaricides for controlling skin affections has some disadvantages such as the development of resistance and toxicities to the environment, feed, and humans [93, 133]. Many studies showed that infestation with *Sarcoptes scabiei* could induce a degree of protective immunity in different species of animals [134–136]. Accordingly, vaccination may be a good alternative to therapy for mange control [137]. Unfortunately, there is no available commercially effective vaccine currently against mange. The research in this area is very limited [138]. Some studies used protein-based candidates of mites as vaccines. Rabbit's vaccination with *Sarcoptes scabiei* tropomyosin allergen showed reduction in lesions' size; however, the vaccine did not completely treat sarcoptic mange [139]. In addition, recombinant antigens such as Ssag1, Ssag2 [140], and *Sarcoptes scabiei* glutathione S-transferase [141] were used as vaccines, but they did not completely protect the animals against *Sarcoptes scabiei* infestation. Tarigan [137] explained this incomplete vaccination protection against mange by the denaturation or degradation of protective antigens and their low abundance or low immunoprotection. Casais et al. [142] demonstrated that immunodominant antigens (Ss*λ*15 and Ss*λ*20ΔB3) that were used as recombinant proteins were not effective as a vaccine against *Sarcoptes scabiei* in rabbits. Although the vaccination with both antigens induced high levels of humoral immunoglobulins (Ig)G and IgE and reduced mite density, this vaccine showed no clinical improvement in the skin lesion scores. Developed recombinant proteins containing purified rSs chitinase-like proteins (CLP)5 and rSs CLP12 subunit cocktail vaccine against *Sarcoptes scabiei* in rabbits induced a strong immune response and significantly decreased the parasite load in the host [64, 65, 143].

## 7. Conclusion

The rabbit farming industry is subject to several challenges that make it difficult to manage. One of the main constraints that still encounters rabbit farming is the mange disease. Many strategies have been applied to control mange in rabbits. The application of restricted hygienic measures surrounding the animals is crucial to prevent such infestation. Humans also must take all precautions during handling rabbits to avoid zoonotic transmission. Searching for new types of acaricides or even developing vaccines should be taken into consideration in future research work.

## Figures and Tables

**Table 1:** Clinical pictures of different mite species infest rabbits.

Species of mite	Clinical picture	Reference(s)
*Psoroptes cuniculi*	Severe skin inflammation, pruritus, erythema, and exudation, as well as presence of crusts, flaky scales, scabs, sores, and ulceration of the inner side of the pinnae and on the external ear canal, were noticed	[33]
	Excessive secretion of red or brownish waxy material in one or both ears was seen. The hypersensitivity reaction of rabbits to the antigenic material of mite could be observed as severe restlessness, irritation, itching and scratching, head shaking, drooping of ears, and foul-smelling discharges from the external ear canal	[34]
	Severe cases could manifest meningitis, which may be fatal when complicated by secondary bacterial infections	[15]
	In the late and chronic stage of infestation, affected rabbits may present anorexia, loss of body weight, growth retardation, emaciation, lethargy, and finally death. Anorexia results from nausea and dizziness caused by ear infestation	[35]
	The adult mite invades the ear epidermis and sucks the lymph causing severe inflammation of the tissues with swelling and the serum exudate coalesces to form crusts	[3, 34]

*Sarcoptes scabiei var. cuniculi*	Skin scabies appears as diffuse erythema, thickening, wrinkling, crust formation, scale production, extensive hyperkeratosis, and alopecia around the ear's pinna, nose, lips, face, legs, abdomen, perianal region, and genitalia	[36]
	Animals show itching, purities, and pyodermatitis	[37]
	Infested rabbits become anorexic and cachectic and then die. High mortalities may reach 22.2% in infested kids	[30]
	Debilitated animals show high mortalities with immunosuppression	[38]

*Cheyletiella parasitovorax*	Crusts, scales, and alopecia of the affected area of the skin were detected	[39]

*Notoedres cati cuniculi*	Formation of scabs with inflammation of ear pinnae, lips, around eyes, nose, face, neck, dorsal back, forelimbs, and around genitalia was observed	[40]

*Psoroptes ovis*	Skin lesions were similar to those of *Psoroptes cuniculi*	[9]

## Data Availability

The data used to support the review are available from the author upon request.
